# Fasting and Nutrition as Promising Treatment Strategies for Women with Rheumatoid Arthritis in Transitional Hormonal Stages

**DOI:** 10.3390/nu18040580

**Published:** 2026-02-10

**Authors:** Bérénice Hansen, Evdokia Alvanou, Maria Angeliki S. Pavlou, Paul Wilmes, Jochen G. Schneider

**Affiliations:** 1 Luxembourg Centre for Systems Biomedicine, University of Luxembourg, L-4365 Esch-sur-Alzette, Luxembourg; 2 Centre Hospitalier du Nord, 120 Av. Lucien Salentiny, 9080 Ettelbruck, Luxembourg; 3 Department of Life Sciences and Medicine, University of Luxembourg, L-4365 Esch-sur-Alzette, Luxembourg; 4 Department of Internal Medicine II, Faculty of Medicine, Saarland University Hospital, Saarland University, 66421 Homburg, Germany

**Keywords:** fasting, nutrition, rheumatoid arthritis, autoimmune diseases, chronic diseases, intermittent fasting

## Abstract

Rheumatoid arthritis (RA) is a systemic and chronic autoimmune disease affecting about 1% of the global population, with a higher prevalence in women. Its treatment has been improved greatly over the past 30 years but there is no definitive cure available, and another unmet need exists for transitional hormonal stages such as pregnancy or menopause, which spurs the need to research new therapy options. In recent years, dietary interventions, particularly fasting and plant-based nutrition, have gained attention for their potential to alleviate RA symptoms. Fasting has been shown to reduce systemic inflammation, promote autophagy, and modulate immune cell activity, possibly leading to decreased joint pain and swelling. Nutritional strategies, such as anti-inflammatory and plant-based diets, have been shown to impact the gut microbiome and potentially support weight management, improve metabolic health, and reduce oxidative stress, all of which might contribute to better RA disease outcomes. Although the precise mechanisms remain under investigation, these approaches offer promising complementary strategies for enhancing RA management and improving patients’ quality of life. This review explores the preventive and therapeutic potential of fasting and nutrition in RA, and their possible application in the context of hormonal fluctuations and transitional stages during a women’s life.

## 1. Introduction

Modern healthcare has increasingly improved in terms of novel drugs and pharmaceutical advances, leading to significant improvements in lifespan and quality of life. However, side effects are also common, and treatment strategies are not always as efficient as desired [1,2]. Lifestyle adaptations and disease prevention rather than treatment, e.g., dietary strategies, are greatly underappreciated [3]. The typical Western diet and lifestyle have contributed to a rise in non-communicable diseases (NCDs), including autoimmune diseases (AIDs), with more patients depending on pharmacological treatments [4,5]. In AIDs, tissue damage leads to disruption of functions of the affected organs [6]. Rheumatoid arthritis (RA) is a systemic and chronic inflammatory AID [7]. The prevalence of RA is about 0.5 to 1.1% of the general population of industrialised countries, with a lower incidence in Southern Europe, and a generally higher prevalence among women, with the ratio being 2-3/1 (1a 3a) [8].

RA most commonly presents with articular inflammation, which can follow three distinct clinical patterns: (1) the most prevalent form is insidious onset of synovitis affecting the small joints of the extremities; (2) a subacute onset, which involves similar localisation but is accompanied by more pronounced systemic symptoms; and (3) a less frequent variation characterised by acute onset of monoarticular disease. RA mostly manifests in genetically predisposed people, such as in those with a HLA-DR4-positive genotype, who develop an immune response to post-translational modified proteins. A very common post-translational modification is citrullination of proteins in mammals, which is promoted by smoking and oral infections. The subclinical phase of the disease can last several years. It may lead to inflammation of the synovial membrane of the joints with increased production of metalloproteinases that cause cartilage degradation, bone loss, and marginal erosions of the joints. Besides the articular features, RA can have accompanying hematologic, ocular, pulmonary, cardiac, renal, and cutaneous manifestations. It is often associated with cardiovascular diseases, mood disturbances, fibromyalgia, and other comorbidities [7,9].

Various treatment strategies, such as non-steroidal anti-inflammatory agents (NSAIDs), corticosteroids, and disease-modifying anti-rheumatic drugs (DMARDs) are available. However, these drugs have significant inconveniences [9]. NSAIDs and corticosteroids are commonly used during acute outbreaks to rapidly relieve symptoms, but potentially cause gastrointestinal complications, cardiovascular risks, and immune suppression. DMARDs, used in long-term treatment strategies, vary in efficacy and often require prolonged use before therapeutic benefits are manifested [8,9].

Despite these different treatment options, responsiveness is not always obtained and only a small percentage achieve sustained remission [9,10,11]. Additionally, these medications are often unsuitable for pregnant women, especially in the third trimester, further emphasising the urgent need for alternative treatment approaches, such as nutrition and lifestyle interventions [9].

Lifestyle factors such as nutrition, exercise, sleep, and stress have been increasingly recognised as key players in health and well-being [12]. Specifically nutritional intervention strategies, like dietary composition, combined with meal timing, have been of interest in NCD prevention and treatment. Plant-based diets and fasting have been previously reported to show promising beneficial effects in RA; however, research is sparse and understanding of precise mechanisms is lacking [13]. Conversely, Western dietary patterns, characterised by high intake of red meat, refined sugars, and saturated fats, have been linked to a higher risk of RA [5]. Additionally, emerging evidence suggests that time-restricted eating (TRE), a form of intermittent fasting (IF), may influence inflammation and immune regulation in RA [14]. IF is defined as repetitive fasting periods lasting up to 48 h each, and TRE is a dietary regimen where the consumption of food is limited to a defined period of time during a 24 h window, resulting in a daily fasting window of at least 14 h [15].

## 2. Rheumatoid Arthritis During Critical Life Phases of Women

As previously mentioned, the RA incidence is higher in woman than in men, which is generally the case for AIDs. Several studies have tried to explore sex discrepancy, but without coming to a conclusive outcome. One hypothesis is that sex hormones might play a key role in the higher prevalence of AIDs in women [16]. Different forms of oestrogen act via their specific oestrogen receptor (ER)-alpha and ER-beta, which are also present on immune cells such as dendritic cells, B cells, and T cells [6]. Through these specific ERs, oestrogens can increase antibody production in females by promoting activation and class switch recombination of B cells [17].

Another hypothesis for the higher AID prevalence in women is linked to the X-chromosomes, as the prevalence for certain AIDs has a higher occurrence in Klinefelter (XXY) and Triple X (XXX) syndromes, while conditions like Turner syndrome (XO) show a lower risk [18]. A review published in 2023 on the higher prevalence of lupus in women explains this by three potential hypotheses: (1) non-random X-chromosome inactivation, (2) incomplete silencing of specific genes on the X-chromosome, (3) or the potential activation of the immune system by the X-Inactive-Specific Transcript ribonucleoprotein complex [18]. This higher prevalence in females introduces unique challenges in the treatment of AIDs, as women face hormonal fluctuations across their lifetime. The hormonal fluctuations across the menstrual cycle, pregnancy, and menopause are often accompanied by changes in immune function and inflammation and significantly impact the disease symptoms and the patient’s well-being [19]. While higher oestrogen levels, as seen during pregnancy, may temporarily attenuate inflammation, decreased oestrogen levels during menopause are associated with increased T cell activation, including enhanced Th17 response [20]. The menstrual cycle has a profound impact on pain perception, and especially in the case of premenstrual syndrome (PMS), many women report increased joint pain [21]. Both oestrogen decrease and ageing have been shown to independently contribute to the worsening of RA, suggesting that hormonal and age-related changes synergistically influence disease progression in women.

Another level of complexity is added in the case of pregnancy, because as previously mentioned, most RA-specific treatments are counter-indicated during this time [22]. Conventional NSAIDs should not be used during the third trimester, potentially leading to an early closure of arteriovenous ductus [23]. While steroid use might be mostly safe due to a minimal crossing of the placental barrier, the use of several DMARDs, such as methotrexate and leflunomide, is contraindicated. Although various drug therapies do not seem to have a noxious effect on the foetus, knowledge is very limited and the current guideline recommends women to avoid pregnancy while under specific RA treatment [23]. Although disease severity might be alleviated or completely suppressed during pregnancy in some cases, especially during the third trimester, relapses are increased postpartum, and not all women experience similar improvements during gestation [6]. PubMed research found only three clinical trials targeting pregnant women with rheumatoid arthritis. The first article by Meade et al. from 2015 focusses on motherhood decision making, exposing the existing complications of the condition, proposing a “Motherhood decision aid” to at least inform the patients of their treatment options [24], however, without offering additional solutions. A second publication tried to mimic the increased levels of alpha-fetoprotein (AFP), correlating with a tendency of remission during the third trimester of pregnancy, by administering a non-glycosylated, recombinant version of AFP to non-pregnant patients with RA. The outcome was positive, offering potential therapeutic strategies for patients with RA in general, missing the opportunity to focus on treatments for pregnant women [25]. The third study in relation to pregnancy and RA had a similar approach. As pregnancy is known to have a protective effect on Th1-mediated AIDs in some cases, oral oestriol treatment was injected in patients suffering from multiple sclerosis leading to an immunomodulatory effect [26]. The previous studies suggest an improvement in RA symptoms during pregnancy, potentially suggesting no need for adapted treatment strategies. However, Jethwa et al. reported that 40% of patients did not experience improved disease activity, and additionally, 46.7% experienced postpartum flares [27]. This emphasises the need for alternative treatment strategies during pregnancy.

Another important hormonal transitional phase for women is perimenopause and menopause. This transition is a disruptive process which can last for over a decade and cause severe symptoms in the majority of women [28]. During this process, ovarian reproductive function gradually ceases, leading to an end of the menstrual cycle. However, during perimenopause, the ovarian function fluctuates greatly, inducing a range of symptoms [29]. Symptoms can range from vasomotor symptoms, mood disruption, temporary cognitive dysfunction, genitourinary symptoms, and others, severely reducing the quality of life of the affected women [28]. Common treatment includes hormonal replacement therapy (HRT). A study performed by the Women’s Health Initiative reported that joint pain or stiffness were common symptoms in menopausal women and a higher symptom relief could be achieved by HRT, although differences were only modest [30]. Although proven to be useful in menopause-specific symptom relief, a combination with RA treatments is complex and needs careful consideration [31].

Lifestyle factors are of great interest here. A randomised control trial investigated the outcomes of an IF regimen over 8 weeks in overweight and obese postmenopausal women with RA [32]. The study found that IF significantly improved body mass index, disease activity scores, and quality of life. Interestingly, certain inflammatory and oxidative stress markers did not reveal significant changes, suggesting that the benefits of IF in this population may be primarily linked to weight loss and physical improvements rather than direct modulation of inflammation or oxidative stress [32]. However, in the IF group, a significant decrease in the neutrophil-to-lymphocyte ratio was observed as well as in the malondialdehyde levels, a well-described marker of oxidative stress [14]. Finally, participants showed favourable changes in liver enzyme profiles, suggesting improved liver function [14].

Taking into account these multiple challenges in women’s health, dietary strategies such as the mediterranean diet (MD) and fasting offer promising, non-pharmacological options. Studies suggest these approaches not only reduce RA activity but also improve general well-being, including PMS and menopause-related symptoms [33]. IF strategies are especially promising as they minimise the risk of malnutrition. IF strategies include patterns such as TRE, where the eating window in a 24 h frame is typically reduced, followed by at least 14 h of fasting. Another strategy is a fasting-mimicking diet (FMD) [15], defined as periodic fasting periods of 3–7 days involving a reduced kcal intake of a low-protein, low-carbohydrate, and high-fat diet, designed to induce fasting-like metabolic states while reducing the risk of malnourishment [34,35].

## 3. Alternative Treatment Strategies for Rheumatoid Arthritis

### 3.1. The Role of the Gut Microbiome in Rheumatoid Arthritis

Dietary interventions are gaining interest due to their potential impact on the GMB, a crucial player in immune regulation and systemic inflammation. Dysbiosis, an imbalance in GMB composition, has been identified as a key factor in RA pathogenesis [36]. Several significant differences in patients with RA compared to healthy controls have been previously reported, such as decreases in *Bacteroidota* and increases in *Firmicutes* and *Pseudomonadota,* including, amongst others, *Porphyromonas gingivalis* and *Aggregatibacter actinomycetemcomitans* [37,38]. Microbial enzymes have also been reported to enhance or reduce drug efficacy, while drugs, in turn, can alter microbial composition, influencing host responses. However, the impact of GMB–drug interactions on treatment efficacy remains unclear [39]. Notably, ex vivo studies showed that residual methotrexate (MTX) levels in distal gut samples from RA patients correlated with future treatment response, suggesting a direct role of the microbiome in MTX metabolism [40]. This is of particular interest considering that some studies suggest GMB fluctuates across the menstrual cycle, as well as that there is an impact of oral contraceptives on the GMB [41]. However, the results are conflicting. An in vitro model by Leao et al. shows a significant influence of sex hormones on microbiome structure and diversity, including specific shifts induced by hormonal fluctuations across the menstrual cycle, including increases at the phylum level in *Bacteroidota* and decreases in *Bacillota* and *Pseudomonadota* [42]. Some studies looked at the effect of oral contraceptives on the GMB. Terrazas et al. report an impact of oral contraceptives on the a-diversity, while Brit et al. report no difference in GMB composition, but find that the ß-diversity differs between the control group and the oral contraception group [41,43]. Krog et al. find no change in the GMB whatsoever, neither in the oral contraceptive nor the control group, and only report changes in vaginal and oral microbiome composition [44]. When it comes to pregnancy, the literature is even more limited. Only two clinical trials on GMB changes have been identified, both in relation to dietary patterns. These studies report changes over the course of the pregnancy in the GMB composition, however, only in association with different dietary interventions, such as a mediterranean diet (MD) and a vegetarian diet [45,46]. Natural GMB fluctuations without dietary intervention have not been reported yet. Similarly for menopause, while twenty-one clinical trials analysing different interventions, including probiotics, prune, and blackcurrant supplementation, have been published, no studies on long-term GMB changes during menopause were identified [47,48].

Besides a potential hormonal influence, factors like stress, sleep, physical activity, and nutrition are influential modulators of GMB composition [49]. Western diets, characterised by high sugar, high fat, and processed food intake, are associated with increased gut permeability, reduced microbial diversity, and higher systemic inflammation [50]. Conversely, diets such as the MD, rich in plant-based foods and fibre, promote beneficial microbial shifts and anti-inflammatory effects [3,5]. The TASTY trial f.ex. focuses on a traditional MD enriched with fermented foods, implemented for 12 weeks. The study is aiming to evaluate the effects of this dietary intervention on gut microbiota composition, intestinal barrier integrity, and clinical outcomes associated with RA [51].

### 3.2. Fasting Interventions and Their Benefits in Rheumatoid Arthritis

**Figure 1:**
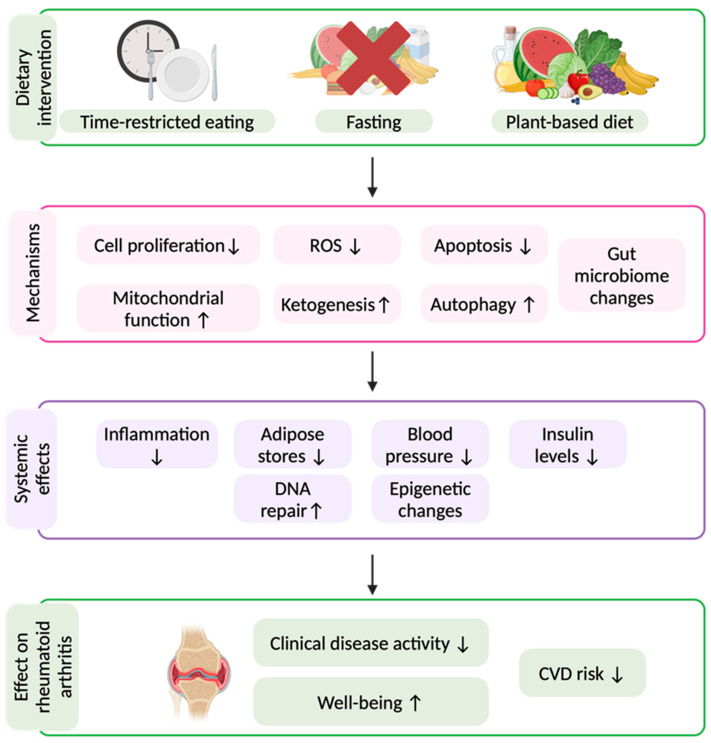
Possible beneficial effects of fasting and dietary interventions on rheumatoid arthritis. ↑: increase, ↓: reduction. ROS: Reactive Oxygen Species, CVD: cardiovascular disease. Created in Biorender, B Hansen 2025.

While traditional dietary patterns emphasise food composition, meal timing is also emerging as a critical factor in metabolic and immune regulation [52]. IF, including TRE as well as FMDs, has gained immense popularity, as it is more easily accessible than fasting protocols such as prolonged fasting (PF), a continuous fasting period of more than 4 days with a kcal intake of max. 350 kcal/day, which requires medical supervision [15]. Fasting modulates metabolism by reducing insulin levels, increasing ketogenesis, altering bile acid secretion, and suppressing pro-inflammatory pathways [53]. Key mechanisms include enhanced autophagy and mitochondrial function and reduced oxidative stress (Figure 1) [53].

In a recent study, RA patients underwent bowel cleansing and then followed a 7-day period of fasting. This protocol led to significant immunological and clinical improvements. Notably, there was a marked reduction in monocyte turnover and a decline in pro-inflammatory cytokines. These changes were accompanied by a significant reduction in disease activity scores, with patients showing clinical improvement and some achieving full remission. Additionally, serum levels of IL-6 and zonulin, a marker of gut permeability, were significantly decreased [54].

The NutriFast trial, conducted at the Charité in Germany, showed that both 1 week of PF followed by 11 weeks of a plant-based diet (PBD) as well as 12 weeks of AD without prior fasting lead to significant improvements in patients with RA [55,56]. Decreased clinical disease activity and improved well-being in RA were accompanied by improvements in cardiovascular risk factors [57]. The results are promising but only observed over the course of 12 weeks. The ExpoBiome study, however, follows patients undergoing one week of PF continued by a maintenance diet of TRE, a form of IF, for 12 months. The results of this study show, amongst others, that PF followed by TRE leads to a sustained decrease in activity, lower BMI, and overall increased well-being for at least 12 months [58,59]. Additionally, the above-mentioned studies portray feasibility and high adherence of the patients with RA to the different fasting regimens. In-depth analyses will focus on the gut microbiome composition and changes as well as on alternations in metabolite production and circulation, such as bile acid and lithocholic acid. The latter has been shown to activate, amongst others, AMPK, a potential key player in fasting-mediated health benefits [60,61]. It needs to be noted that the ExpoBiome trial has one arm of patients with RA, and a similar set-up with a higher patient number and an additional control group would be essential for a follow-up study.

While research is still ongoing, emerging evidence suggests that IF may offer beneficial effects for autoimmune and inflammatory conditions like RA, primarily by reducing inflammation and modulating immune responses [62].

### 3.3. Other Dietary Intervention Studies

Recent and ongoing studies focus on the impact of dietary interventions such as a PBD or an AD and their effect on RA. A systematic review of PBD on patients with RA suggests that the observed beneficial effects may stem from the diet’s anti-inflammatory properties and antioxidant content, which together could enhance GMB, reduce immune reactivity to food allergens, and lower intestinal inflammation often associated with meat consumption [63].

In the MADEIRA randomised control trial, women with RA underwent a three-month intervention that combined a personalised isocaloric MD plan with the encouragement of physical activity, all assisted by a clinical decision support system platform. The intervention group demonstrated significantly higher adherence to the MD compared to the control group. These women also showed notable reductions in disease activity scores, along with improvements in dietary intake, physical activity levels, body weight, blood glucose levels, and serum 1,25-dihydroxyvitamin D concentrations. These results suggest that this combined protocol may lead to meaningful improvements in disease activity and cardiometabolic health in women with RA [64].

Another approach concerns the Autoimmune Protocol (AIP) diet that has been implemented in several studies in patients with autoimmune diseases. The AIP diet is a patient-specific tailored elimination diet designed to identify and remove foods that may provoke immune reactions due to a dysfunction in the gut barrier, contributing to inflammation and symptoms linked to autoimmune conditions. It involves three key stages: elimination of all potential foods that trigger an immune reaction, reintroduction of the reaction-free foods, and long-term maintenance of the individual-appropriate diet [65]. The initial finding suggest that the AIP may serve as a promising complementary approach in the management of RA, requiring further clinical investigation to confirm its efficacy [66].

### 3.4. Fasting and Dietary Intervention Studies in Transitional Hormonal Stages

Research into female-specific conditions such as menstruation, including PMS and PCOS, as well as pregnancy and menopause, has only gained attention relatively recently. As a result, the scientific landscape in these areas remains comparatively sparse. While no clinical trials on fasting and PMS symptoms are found, several publications suggest improved symptom outcomes for fasting interventions, mostly TRE, in PCOS and postmenopause, including weight reduction and improvements in hyperandrogenaemia, body composition, and several metabolic parameters in obese women [67,68,69]. Similar improvements have been reported for dietary interventions such as a MD combined with other lifestyle changes, including increased physical activity [70,71].

## 4. Discussion

RA disproportionately affects women, particularly during critical hormonal transitions such as pregnancy, postpartum, and menopause. These phases are often accompanied by shifts in immune function and inflammatory responses, underlining the need for prevention and treatment strategies that are specifically tailored to women’s health. For instance, the interplay between oestrogen levels and immune modulation has been shown to influence RA onset and activity, with symptoms often improving during pregnancy and flaring postpartum. The symptom alleviation could potentially be increased by applying diverse lifestyle changes, such as TRE and adapted dietary intake. To avoid any kind of nutrient deficiencies during pregnancy, the focus should ideally be on IF or short-term fasting protocols, such as brief fasting periods of 2–4 days, which may exert metabolic and immunomodulatory effects without sustained caloric deprivation. Any consideration of such strategies during pregnancy would require strict clinical oversight and individualised assessment to ensure maternal and foetal nutritional adequacy. Unfortunately, nutrition is still underappreciated. A strong emphasis on pharmacological knowledge in medical education, while essential, sometimes overshadows the role of nutrition in clinical practice. Combined with society’s tendency toward a preference for quick solutions, this can limit the active application of diverse dietary and lifestyle strategies for symptom alleviation.

A combined approach that integrates pharmacological treatments with lifestyle interventions could significantly enhance the management of RA and other NCDs. While medications such as DMARDs help control inflammation and slow disease progression, complementary dietary and lifestyle strategies can further reduce symptoms and improve overall well-being and offer opportunities for patients not able to profit from DMARDs. By considering both pharmacological solutions and evidence-based lifestyle modifications, patients may achieve better long-term outcomes, potentially requiring lower medication doses. Additionally, as mentioned above, several drugs can interfere with GMB composition, potentially affecting immunity and even medication efficacy. This is of particular importance for women, whose GMB can fluctuate in response to menstrual cycles, pregnancy, and hormonal therapies. A combined approach, incorporating targeted nutritional strategies, as well as the possible inclusion of probiotics, could help support microbiome balance and mitigate unintended side effects. Such a multifaceted strategy underscores the potential for medicine and lifestyle to work synergistically rather than independently.

The establishment of standardised, sex-specific guidelines combined with nutritional education could be of high interest, allowing patients to make informed dietary choices and thereby reducing their disease activity. Adequate nutritional education for both patient and caretaker could avoid any potential risks induced by dietary changes and restrictive food intake. Additionally, women need to be informed and prepared for potential transient side effects of fasting, including headaches, bad breath, irritability, and tiredness, amongst others. Besides targeting several issues raised above, dietary interventions are empowering and accessible for patients as they reduce their dependency and increase their autonomy. For women managing RA during pregnancy or menopause, these interventions could offer safer, non-pharmacological tools that align with both of their physiological needs.

The outlined potential of combined treatment strategies emphasises the need for more high-quality research, focusing on understanding the complex underlying mechanisms of dietary interventions, such as fasting, in RA. Studies mentioned in this article often come with limitations such as small patient groups, single-arm setups, and a short intervention period. Therefore, future research needs to focus on long-term sustainability of fasting benefits, sex-specific impacts, personalised nutritional interventions, and GMB interactions, and subsequently pave the way for a higher visibility and recognition of dietary interventions into clinical practice.

To conclude, nutrition represents a powerful, underutilised tool in RA management, particularly for women navigating distinct hormonal life stages. With growing evidence linking diet, the microbiome, and immune function, integrative, personalised treatment plans must become a non-negotiable component of patient-centred care in rheumatology.

## Data Availability

The dataset from this study is available from the authors upon reasonable request.

## References

[1] Genovese M.C., Fleischmann R., Combe B., Hall S., Rubbert-Roth A., Zhang Y., Zhou Y., Mohamed M.F., Meerwein S., Pangan A.L. (2018). Safety and efficacy of upadacitinib in patients with active rheumatoid arthritis refractory to biologic disease-modifying anti-rheumatic drugs (SELECT-BEYOND): A double-blind, randomised controlled phase 3 trial. Lancet.

[2] Fleischmann R., Meerwein S., Charles-Schoeman C., Combe B., Hall S., Khan N., Carter K.M., Camp H.S., Rubbert-Roth A. (2024). Efficacy and safety of upadacitinib in patients with rheumatoid arthritis and inadequate response or intolerance to biological treatments: Results through 5 years from the SELECT-BEYOND study. RMD Open.

[3] Desai M.S., Seekatz A.M., Koropatkin N.M., Kamada N., Hickey C.A., Wolter M., Pudlo N.A., Kitamoto S., Terrapon N., Muller A. (2016). A Dietary Fiber-Deprived Gut Microbiota Degrades the Colonic Mucus Barrier and Enhances Pathogen Susceptibility. Cell.

[4] Budreviciute A., Damiati S., Sabir D.K., Onder K., Schuller-Goetzburg P., Plakys G., Katileviciute A., Khoja S., Kodzius R. (2020). Management and Prevention Strategies for Non-communicable Diseases (NCDs) and Their Risk Factors. Front. Public Health.

[5] Gioia C., Lucchino B., Tarsitano M.G., Iannuccelli C., Di Franco M. (2020). Dietary Habits and Nutrition in Rheumatoid Arthritis: Can Diet Influence Disease Development and Clinical Manifestations?. Nutrients.

[6] Lasrado N., Jia T., Massilamany C., Franco R., Illes Z., Reddy J. (2020). Mechanisms of sex hormones in autoimmunity: Focus on EAE. Biol. Sex Differ..

[7] Scherer H.U., Häupl T., Burmester G.R. (2020). The etiology of rheumatoid arthritis. J. Autoimmun..

[8] Ben Mrid R., Bouchmaa N., Ainani H., El Fatimy R., Malka G., Mazini L. (2022). Anti-rheumatoid drugs advancements: New insights into the molecular treatment of rheumatoid arthritis. Biomed. Pharmacother..

[9] Fatima T., Zhang Y., Vasileiadis G.K., Rawshani A., van Vollenhoven R., Lampa J., Gudbjornsson B., Haavardsholm E.A., Nordström D., Gröndal G. (2025). Disease activity and treatment response in early rheumatoid arthritis: An exploratory metabolomic profiling in the NORD-STAR cohort. Arthritis Res. Ther..

[10] Chatzidionysiou K., Sfikakis P.P. (2019). Low rates of remission with methotrexate monotherapy in rheumatoid arthritis: Review of randomised controlled trials could point towards a paradigm shift. RMD Open.

[11] Wang Z., Huang J., Xie D., He D., Lu A., Liang C. (2021). Toward Overcoming Treatment Failure in Rheumatoid Arthritis. Front. Immunol..

[12] Magomedova A., Fatima G. (2025). Mental Health and Well-Being in the Modern Era: A Comprehensive Review of Challenges and Interventions. Cureus.

[13] Sköldstam L., Larsson L., Lindström F.D. (1979). Effect of fasting and lactovegetarian diet on rheumatoid arthritis. Scand. J. Rheumatol..

[14] Tavakoli A., Akhgarjand C., Ansar H., Houjaghani H., Khormani A., Djafarian K., Rostamian A., Ranjbar M., Farsani G.M. (2025). The effects of intermittent fasting on antioxidant and inflammatory markers and liver enzymes in postmenopausal, overweight and obese women with rheumatoid arthritis: A randomized controlled trial. Sci. Rep..

[15] Koppold D.A., Breinlinger C., Hanslian E., Kessler C., Cramer H., Khokhar A.R., Peterson C.M., Tinsley G., Vernieri C., Bloomer R.J. (2024). International consensus on fasting terminology. Cell Metab..

[16] Hoffmann J.P., Liu J.A., Seddu K., Klein S.L. (2023). Sex hormone signaling and regulation of immune function. Immunity.

[17] Fairweather D., Beetler D.J., McCabe E.J., Lieberman S.M. (2024). Mechanisms underlying sex differences in autoimmunity. J. Clin. Investig..

[18] Vieira A.A., Almada-Correia I., Inácio J., Costa-Reis P., da Rocha S.T. (2024). Female-bias in systemic lupus erythematosus: How much is the X chromosome to blame?. Biol. Sex Differ..

[19] Desai M.K., Brinton R.D. (2019). Autoimmune Disease in Women: Endocrine Transition and Risk Across the Lifespan. Front. Endocrinol..

[20] Khalaf S., Salman A., Yuosif S. (2025). Review on the Role of Estrogen Hormone in Rheumatoid Arthritis Disease. Yemeni J. Med. Sci..

[21] Hellström B., Anderberg U.M. (2003). Pain perception across the menstrual cycle phases in women with chronic pain. Percept. Mot. Ski..

[22] Partlett R., Roussou E. (2011). The treatment of rheumatoid arthritis during pregnancy. Rheumatol. Int..

[23] Calvo Alén J. (2009). [Management of difficult clinical situations in patients with rheumatoid arthritis: Pregnancy]. Reum. Clin..

[24] Meade T., Dowswell E., Manolios N., Sharpe L. (2015). The motherhood choices decision aid for women with rheumatoid arthritis increases knowledge and reduces decisional conflict: A randomized controlled trial. BMC Musculoskelet. Disord..

[25] Pollard L.C., Murray J., Moody M., Stewart E.J., Choy E.H. (2007). A randomised, double-blind, placebo-controlled trial of a recombinant version of human alpha-fetoprotein (MM-093) in patients with active rheumatoid arthritis. Ann. Rheum. Dis..

[26] Soldan S.S., Alvarez Retuerto A.I., Sicotte N.L., Voskuhl R.R. (2003). Immune modulation in multiple sclerosis patients treated with the pregnancy hormone estriol. J. Immunol..

[27] Jethwa H., Lam S., Smith C., Giles I. (2019). Does Rheumatoid Arthritis Really Improve During Pregnancy? A Systematic Review and Metaanalysis. J. Rheumatol..

[28] Santoro N., Roeca C., Peters B.A., Neal-Perry G. (2021). The Menopause Transition: Signs, Symptoms, and Management Options. J. Clin. Endocrinol. Metab..

[29] Gatenby C., Simpson P. (2024). Menopause: Physiology, definitions, and symptoms. Best Pract. Res. Clin. Endocrinol. Metab..

[30] Gulati M., Dursun E., Vincent K., Watt F.E. (2023). The influence of sex hormones on musculoskeletal pain and osteoarthritis. Lancet Rheumatol..

[31] Sammaritano L.R., Bermas B.L., Chakravarty E.E., Chambers C., Clowse M.E.B., Lockshin M.D., Marder W., Guyatt G., Branch D.W., Buyon J. (2020). 2020 American College of Rheumatology Guideline for the Management of Reproductive Health in Rheumatic and Musculoskeletal Diseases. Arthritis Care Res..

[32] Ranjbar M., Shab-Bidar S., Rostamian A., Mohammadi H., Tavakoli A., Djafarian K. (2025). Effects of intermittent fasting diet in overweight and obese postmenopausal women with rheumatoid arthritis: A randomized controlled clinical trial. Complement. Ther. Med..

[33] Siminiuc R., Ţurcanu D. (2023). Impact of nutritional diet therapy on premenstrual syndrome. Front. Nutr..

[34] Choi I.Y., Piccio L., Childress P., Bollman B., Ghosh A., Brandhorst S., Suarez J., Michalsen A., Cross A.H., Morgan T.E. (2016). A Diet Mimicking Fasting Promotes Regeneration and Reduces Autoimmunity and Multiple Sclerosis Symptoms. Cell Rep..

[35] Caffa I., Spagnolo V., Vernieri C., Valdemarin F., Becherini P., Wei M., Brandhorst S., Zucal C., Driehuis E., Ferrando L. (2020). Fasting-mimicking diet and hormone therapy induce breast cancer regression. Nature.

[36] Dehner C., Fine R., Kriegel M.A. (2019). The microbiome in systemic autoimmune disease: Mechanistic insights from recent studies. Curr. Opin. Rheumatol..

[37] Coradduzza D., Bo M., Congiargiu A., Azara E., De Miglio M.R., Erre G.L., Carru C. (2023). Decoding the Microbiome’s Influence on Rheumatoid Arthritis. Microorganisms.

[38] Looh S.C., Soo Z.M.P., Wong J.J., Yam H.C., Chow S.K., Hwang J.S. (2022). Aggregatibacter actinomycetemcomitans as the Aetiological Cause of Rheumatoid Arthritis: What Are the Unsolved Puzzles?. Toxins.

[39] Fan J., Jiang T., He D. (2023). Advances in the implications of the gut microbiota on the treatment efficacy of disease-modifying anti-rheumatic drugs in rheumatoid arthritis. Front. Immunol..

[40] Artacho A., Isaac S., Nayak R., Flor-Duro A., Alexander M., Koo I., Manasson J., Smith P.B., Rosenthal P., Homsi Y. (2021). The Pretreatment Gut Microbiome Is Associated With Lack of Response to Methotrexate in New-Onset Rheumatoid Arthritis. Arthritis Rheumatol..

[41] Terrazas F., Kelley S.T., DeMasi T., Giltvedt K., Tsang M., Nannini K., Kern M., Hooshmand S. (2025). Influence of menstrual cycle and oral contraception on taxonomic composition and gas production in the gut microbiome. J. Med. Microbiol..

[42] Leao L., Esmail G.A., Miri S., Mottawea W., Hammami R. (2025). In vitro modeling of the female gut microbiome: Effects of sex hormones and psychotropic drugs. Microbiol. Spectr..

[43] Brito J., Grosicki G.J., Robinson A.T., Coburn J.W., Costa P.B., Holmes K.E., Lyon G., Hakonsson Z., Conti F., Galpin A.J. (2025). Hormonal birth control is associated with altered gut microbiota β-diversity in physically active females across the menstrual cycle: A pilot trial. J. Appl. Physiol..

[44] Krog M.C., Hugerth L.W., Fransson E., Bashir Z., Nyboe Andersen A., Edfeldt G., Engstrand L., Schuppe-Koistinen I., Nielsen H.S. (2022). The healthy female microbiome across body sites: Effect of hormonal contraceptives and the menstrual cycle. Hum. Reprod..

[45] Barrett H.L., Gomez-Arango L.F., Wilkinson S.A., McIntyre H.D., Callaway L.K., Morrison M., Dekker Nitert M. (2018). A Vegetarian Diet Is a Major Determinant of Gut Microbiota Composition in Early Pregnancy. Nutrients.

[46] Selma-Royo M., Crispi F., Castro-Barquero S., Casas I., Larroya M., Genero M., Paules C., Benitez L., Youssef L., Pascal R. (2025). Effects of Mediterranean diet or Mindfulness-Based Stress Reduction during pregnancy on maternal gut and vaginal microbiota: A subanalysis of the Improving Mothers for a better PrenAtal Care Trial BarCeloNa (IMPACT BCN) trial. Am. J. Clin. Nutr..

[47] Nosal B.M., Thornton S.N., Darooghegi Mofrad M., Sakaki J.R., Mahoney K.J., Macdonald Z., Daddi L., Tran T.D.B., Weinstock G., Zhou Y. (2024). Blackcurrants shape gut microbiota profile and reduce risk of postmenopausal osteoporosis via the gut-bone axis: Evidence from a pilot randomized controlled trial. J. Nutr. Biochem..

[48] Simpson A.M.R., De Souza M.J., Damani J., Rogers C., Williams N.I., Weaver C., Ferruzzi M.G., Chadwick-Corbin S., Nakatsu C.H. (2022). Prune supplementation for 12 months alters the gut microbiome in postmenopausal women. Food Funct..

[49] Donoso F., Cryan J.F., Olavarría-Ramírez L., Nolan Y.M., Clarke G. (2023). Inflammation, Lifestyle Factors, and the Microbiome-Gut-Brain Axis: Relevance to Depression and Antidepressant Action. Clin. Pharmacol. Ther..

[50] Malesza I.J., Malesza M., Walkowiak J., Mussin N., Walkowiak D., Aringazina R., Bartkowiak-Wieczorek J., Mądry E. (2021). High-Fat, Western-Style Diet, Systemic Inflammation, and Gut Microbiota: A Narrative Review. Cells.

[51] Charneca S., Hernando A., Almada-Correia I., Polido-Pereira J., Vieira A., Sousa J., Almeida A.S., Motta C., Barreto G., Eklund K.K. (2025). TASTY trial: Protocol for a study on the triad of nutrition, intestinal microbiota and rheumatoid arthritis. Nutr. J..

[52] Heddes M., Altaha B., Niu Y., Reitmeier S., Kleigrewe K., Haller D., Kiessling S. (2022). The intestinal clock drives the microbiome to maintain gastrointestinal homeostasis. Nat. Commun..

[53] Hansen B., Roomp K., Ebid H., Schneider J.G. (2024). Perspective: The Impact of Fasting and Caloric Restriction on Neurodegenerative Diseases in Humans. Adv. Nutr..

[54] Häupl T., Sörensen T., Smiljanovic B., Darcy M., Scheder-Bieschin J., Steckhan N., Hartmann A.M., Koppold D.A., Stuhlmüller B., Skriner K. (2023). Intestinal Microbiota Reduction Followed by Fasting Discloses Microbial Triggering of Inflammation in Rheumatoid Arthritis. J. Clin. Med..

[55] Hartmann A.M., Dell’Oro M., Spoo M., Fischer J.M., Steckhan N., Jeitler M., Häupl T., Kandil F.I., Michalsen A., Koppold-Liebscher D.A. (2022). To eat or not to eat—An exploratory randomized controlled trial on fasting and plant-based diet in rheumatoid arthritis (NutriFast-Study). Front. Nutr..

[56] Hartmann A.M., Dell’Oro M., Kessler C.S., Schumann D., Steckhan N., Jeitler M., Fischer J.M., Spoo M., Kriegel M.A., Schneider J.G. (2021). Efficacy of therapeutic fasting and plant-based diet in patients with rheumatoid arthritis (NutriFast): Study protocol for a randomised controlled clinical trial. BMJ Open.

[57] Hartmann A.M., D’Urso M., Dell’Oro M., Koppold D.A., Steckhan N., Michalsen A., Kandil F.I., Kessler C.S. (2023). Post Hoc Analysis of a Randomized Controlled Trial on Fasting and Plant-Based Diet in Rheumatoid Arthritis (NutriFast): Nutritional Supply and Impact on Dietary Behavior. Nutrients.

[58] Hansen B., Laczny C.C., Aho V.T.E., Frachet-Bour A., Habier J., Ostaszewski M., Michalsen A., Hanslian E., Koppold D.A., Hartmann A.M. (2023). Protocol for a multicentre cross-sectional, longitudinal ambulatory clinical trial in rheumatoid arthritis and Parkinson’s disease patients analysing the relation between the gut microbiome, fasting and immune status in Germany (ExpoBiome). BMJ Open.

[59] Hansen B., Villette R., Petrov V., Laczny C., Vahid F., Roomp K., Hanslian E., Liebscher D., Rajput Khokhar A., Jeitler M. (2025). Fasting-Driven Suppression of Disease Activity in Rheumatoid Arthritis. Sci. Food.

[60] Qu Q., Chen Y., Wang Y., Long S., Wang W., Yang H.-Y., Li M., Tian X., Wei X., Liu Y.-H. (2024). Lithocholic acid phenocopies anti-ageing effects of calorie restriction. Nature.

[61] Qu Q., Chen Y., Wang Y., Wang W., Long S., Yang H.Y., Wu J., Li M., Tian X., Wei X. (2024). Lithocholic acid binds TULP3 to activate sirtuins and AMPK to slow down ageing. Nature.

[62] Adawi M., Watad A., Brown S., Aazza K., Aazza H., Zouhir M., Sharif K., Ghanayem K., Farah R., Mahagna H. (2017). Ramadan Fasting Exerts Immunomodulatory Effects: Insights from a Systematic Review. Front. Immunol..

[63] Bostan Z.Z., Şare Bulut M., Özen Ünaldı B., Albayrak Buhurcu C., Akbulut G. (2025). Effect of Plant-Based Diets on Rheumatoid Arthritis: A Systematic Review. Nutr. Rev..

[64] Papandreou P., Gioxari A., Daskalou E., Grammatikopoulou M.G., Skouroliakou M., Bogdanos D.P. (2023). Mediterranean Diet and Physical Activity Nudges versus Usual Care in Women with Rheumatoid Arthritis: Results from the MADEIRA Randomized Controlled Trial. Nutrients.

[65] Pardali E.C., Gkouvi A., Gkouskou K.K., Manolakis A.C., Tsigalou C., Goulis D.G., Bogdanos D.P., Grammatikopoulou M.G. (2025). Autoimmune protocol diet: A personalized elimination diet for patients with autoimmune diseases. Metab. Open.

[66] McNeill J., Zinn C., Mearns G., Grainger R. (2023). What Is the Efficacy of the Autoimmune Protocol (AIP) Diet in People with Rheumatoid Arthritis? A Mixed-Methods Pilot Intervention Study. Med. Sci. Forum.

[67] Deshmukh H., Papageorgiou M., Wells L., Akbar S., Strudwick T., Deshmukh K., Vitale S.G., Rigby A., Vince R.V., Reid M. (2023). The Effect of a Very-Low-Calorie Diet (VLCD) vs. a Moderate Energy Deficit Diet in Obese Women with Polycystic Ovary Syndrome (PCOS)-A Randomised Controlled Trial. Nutrients.

[68] Li C., Xing C., Zhang J., Zhao H., Shi W., He B. (2021). Eight-hour time-restricted feeding improves endocrine and metabolic profiles in women with anovulatory polycystic ovary syndrome. J. Transl. Med..

[69] Jóźwiak B., Domin R., Krzywicka M., Laudańska-Krzemińska I. (2024). Effect of exercise alone and in combination with time-restricted eating on cardiometabolic health in menopausal women. J. Transl. Med..

[70] Tan A., Dunseath G., Thomas R.L., Prior S.L., Bracken R.M., Churm R. (2025). Effect of home-based exercise with or without a Mediterranean-style diet on adiposity markers in postmenopausal women: A randomized-control trial. Physiol. Rep..

[71] Carruba G., Granata O.M., Pala V., Campisi I., Agostara B., Cusimano R., Ravazzolo B., Traina A. (2006). A traditional Mediterranean diet decreases endogenous estrogens in healthy postmenopausal women. Nutr. Cancer.

